# Antithrombotic drug removal with hemoadsorption during off-pump coronary artery bypass grafting

**DOI:** 10.1186/s13019-024-02772-1

**Published:** 2024-04-18

**Authors:** Helmut Mair, Stephanie Ulrich, Dow Rosenzweig, Johannes Goeppl, Christopher Jurma, Ferdinand Vogt, Benedikt Baumer, Frank Vogel, Peter Lamm

**Affiliations:** 1 Department of Cardiac Surgery, Artemed Klinikum München Süd Am Isarkanal 30, 81379 Munich, Germany; 2 Department of Cardiology, Benedictus Krankenhaus Tutzing, 82327 Tutzing, Germany; 3 Department of Cardiac Surgery, Paracelsus Medical University, 90471 Nuremberg, Germany; 4 Department of Anesthesiology, Artemed Klinikum München Süd, 81379 Munich, Germany; 5 Department of Cardiac Surgery, University of Munich, 81377 Munich, Germany

**Keywords:** Hemoadsorption, OPCAB, Antithrombotic removal, Cardiac surgery, Cytosorb, Ticagrelor, Rivaroxaban, NOACs, DOACs

## Abstract

**Background:**

Patients requiring coronary artery bypass grafting (CABG) are often loaded with antithrombotic drugs (AT) and are at an increased risk for perioperative bleeding complications. Active AT removal by a hemoadsorption cartridge integrated in the cardiopulmonary bypass circuit is increasingly used in this setting to reduce bleeding, and herein we describe the extension of this application in patients on AT undergoing off-pump coronary artery bypass (OPCAB).

**Methods:**

Ten patients (80% male; mean age: 67.4 ± 9.2years) were treated with ticagrelor (eight patients), rivaroxaban and ticagrelor (one patient), and rivaroxaban (one patient) prior to OPCAB surgery. AT’s were discontinued one day before surgery in nine patients and on the day of surgery in one patient, and all patients were also on aspirin. The cohort mean EuroSCORE-II was 2.9 ± 1.5%. A hemoadsorption cartridge was integrated into a dialysis device (n=4) or a stand-alone apheresis pump (n=6) periprocedural, for a treatment time of 145 ± 33 min. Outcome measures included bleeding according to Bleeding Academic Research Consortium (BARC)-4 and 24-hour chest-tube-drainage (CTD).

**Results:**

Mean operation time was 184 ± 35 min. All patients received a left internal thoracic artery with a mean of 2.3 ± 0.9 total grafts. One patient had a BARC-4 bleeding event and there were no surgical re-explorations for bleeding. Mean 24-hours CTD was 680 ± 307mL. During follow-up of 19.5 ± 17.0 months, none of the patients died or required further reinterventions. No device-related adverse events were reported.

**Conclusions:**

Hemoadsorption via a stand-alone apheresis pump during OPCAB surgery was feasible and safe. This innovative and new approach showed favorable bleeding rates in patients on antithrombotic drugs requiring bypass surgery.

## Background

Antithrombotic drugs (AT) are first line treatment for acute coronary syndromes, atrial fibrillation and other forms of cardiovascular disease, and their use continues to increase by demonstrating improved clinical outcomes, particularly fewer major adverse cardiac and cerebral events (MACCE) supported by numerous large-scale trials and guidelines [1–3]. Simultaneously, patients (pts) who receive AT and require cardiac surgery are exposed to higher risks for adverse events, and, most concerningly, a significantly increased risk of bleeding especially in urgent surgical interventions [4].

Guidelines support continuation of aspirin during coronary artery bypass grafting (CABG) but require multiple days of washout for other AT’s such as P2Y_12_ inhibitors or direct oral anticoagulants (DOAC) [1]. However, in urgent or emergent situations patients may need to be operated on before the necessary washout period can be completed.

To date, these patients could only be managed with supportive measures such as blood product transfusions, but more recently, active intraoperative AT removal via hemoadsorption has emerged as a novel technique. Previous reports have shown that the use of intraoperative hemoadsorption can significantly reduce bleeding complications in patients on ticagrelor or rivaroxaban undergoing urgent cardiac surgery [5–7]. However, the use of hemoadsorption to remove AT’s during cardiac surgery has been limited so far to on-pump cases only with integration of the device into the cardiopulmonary bypass (CPB) circuit.

We hereby describe a novel approach to integrate a hemoadsorption device into an extracorporeal roller pump used for the removal of ticagrelor and rivaroxaban during off-pump coronary artery bypass (OPCAB) grafting in a case series setting.

## Patients and methods

The current study represents a single institution retrospective observational case series which was performed at the Artemed Clinic Munich South, Germany between 2019 and 2022. Consecutive patients on ticagrelor and/or rivaroxaban with an urgent CABG indication and eligibility for OPCAB surgery due to comorbidity were included.

### Ethical statement

The study was conducted in accordance with the Declaration of Helsinki and the protocol was approved by the Ethics Committee of the University of Munich, Germany (approval number: 21–0198). Patient consent was waived due to the retrospective character of the study and de-identified dataset. In the present analysis, an individualized approach was undertaken to ensure a favorable benefit: risk profile in each patient before proceeding with this intervention during OPCAB surgery and was discussed with all patients during the informed consent process for surgery or was considered as best practice decision during the surgery in the instance of excessive bleeding.

### Inclusion criteria

Inclusion criteria included: (a) urgent cardiac surgery in patients aged 18 years or over, male or female (women of child-bearing potential with a negative pregnancy test at screening) on ticagrelor and/or rivaroxaban and (b) use of the hemoadsorption cartridge (Cytosorb®, CytoSorbents, Princeton, NJ, USA) for AT removal during OPCAB surgery.

### Exclusion criteria

Exclusion criteria included concomitant surgery (e.g. aortic valve surgery etc.), patients on chemotherapy, immunosuppressive medication, other antithrombotic drugs than ticagrelor or rivaroxaban or steroids.

### Anticoagulation protocol

Before surgery, citrate anticoagulation was used when the adsorber was integrated into a dialysis pump to start the hemoadsorption therapy. In cases where the adsorber was inserted in the apharesis pump PUR-01, 5,000IE of heparin were administered to initialize the start of the hemoadsorption therapy. After skin-incision, following sternotomy and left internal thoracic artery (LITA) harvesting, another 15,000IE of heparin were administered (targeted activated clotting time > 300s). After completion of all anastomoses, protamine was given.

### Hemoadsorption therapy

The CytoSorb® adsorber consists of a 300 mL cartridge which contains biocompatible porous polymer beads. These beads bind hydrophobic substances with a molecular size of up to 60 kDa from whole blood by pore capture and its irreversible surface adsorption properties. Intraoperative antithrombotic removal via hemoadsorption allows for on-pump ticagrelor and rivaroxaban removal according to the instructions for use and bears CE-mark approval for this indication. Adsorption is also concentration-dependent and the adsorber is approved to remove cytokines, bilirubin, myoglobin, rivaroxaban and ticagrelor. On the other hand, albumin or coagulation factors are not removed, however, certain drugs, e.g. vancomycin are removed [8]. A detailed list of adsorbed substances is given in Scheier et al. and for specific drugs, therapeutic drug monitoring is recommended [9]. The overall adsorption surface area in one adsorption cartridge is more than 45,000m^2^. The adsorber has been demonstrated to remove antithrombotic medications such as ticagrelor [10] and rivaroxaban [7, 11] and is CE-mark approved to remove these AT’s during CPB. In this present analysis, the adsorber was incorporated into a separate apheresis or dialysis pump during off-pump surgery.

### Set up with dialysis pump

The set-up has been described elsewhere by our group, but in brief: The CytoSorb adsorber was integrated with standard dialysis connectors into an extracorporeal circuit in hemoperfusion mode (multiFiltrate, Fresenius Medical Care, Bad Homburg, Germany) using citrate anticoagulation and was run using standard settings (tubes and hemofilter, Kit Ci-Ca CVVHD 1000, Fresenius Medical Care; dialysate, Ci-Ca Dialysate K2, 2 L/h, Fresenius Medical Care). No ultrafiltration was used. Tubes were connected to a large 12-F, 3-lumen catheter (Arrow International Inc., Reading, PA, USA) and were implanted into the femoral vein of the patient. The blood flow rate of this system was set up to 150 mL/h [12].

### Set up with apheresis pump PUR-01

**Fig. 1:**
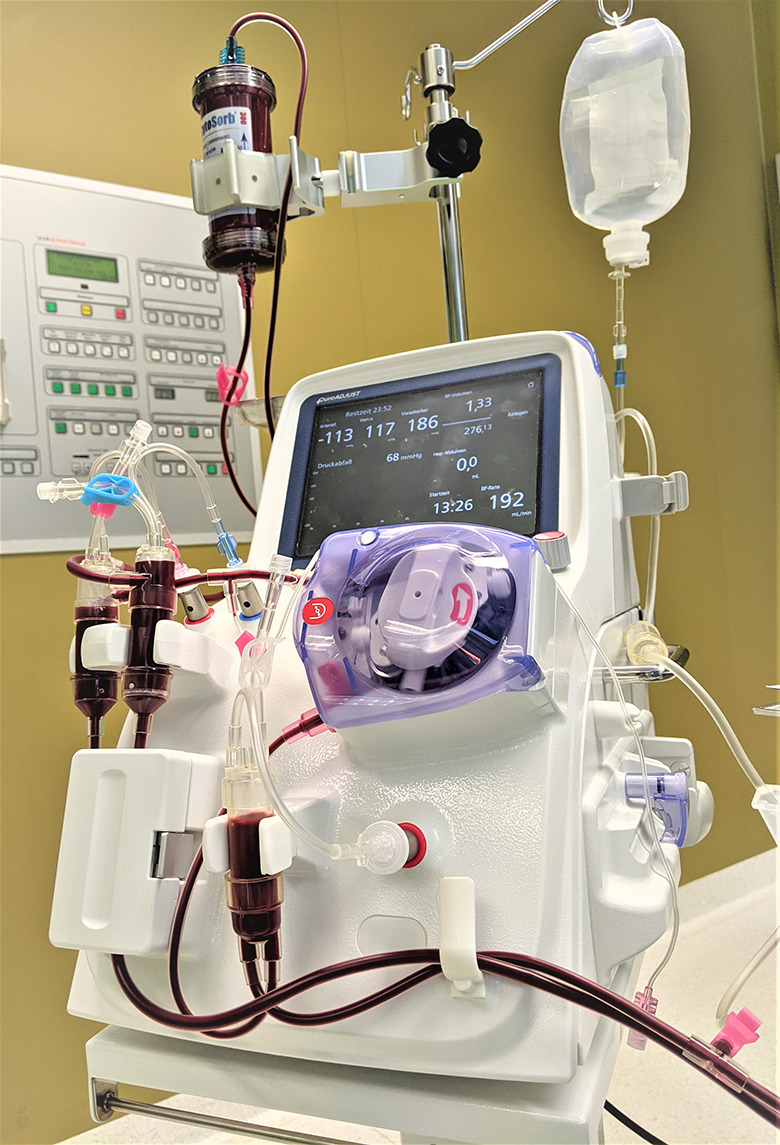
Hemoadsorption device (CytoSorb® 300, CytoSorbents Inc., Princeton, NJ, USA) incorporated in the apheresis machine PUR-01 (Nikkisio Co., Ltd., Tokyo, Japan)

Detailed instructions for use of the PUR-01 pump are described in the “Apheresis Equipment Pure Adjust PUR-01 Operating Instructions” (Manual No. 1055en-R8, 2020-9, Nikkisio CO., Ltd., Tokyo, Japan). In brief: A stand-alone apheresis pump, Pure Adjust PUR-01 (Nikkisio Co., Ltd., Tokyo, Japan) was used (Fig. 1). The apheresis machine has the function of a direct hemoperfusion pump. The machine was equipped with a venous blood tubing line set (ABT-023P Series, Nikkisio Co., Ltd., Tokyo, Japan). The lines of the device were connected to a 12 F and 3-lumen high-flow catheter (Arrow International Inc., Reading, PA, USA) inserted into the cervical or femoral vein of the patient. With the start of PUR-01, a 5000 I.E. single injection of heparin was given. During hemoadsorption the blood flow rate was set up to 200mL/min.

### OPCAB-procedure

Ticagrelor or rivaroxaban was stopped before surgery. The first four patients had the high-flow catheter implanted under local anesthesia prior to surgery, on the ICU. Hemoadsorption was initiated approximately one hour before the patient was transferred to the operation room and was continued for approximately 1 to 1.5 h during the operation for a total treatment time of 145 ± 33 min, to eliminate the AT. The following 6 patients received a 12 F, 3-lumen high-flow catheter implanted into the right femoral or cervical vein after initiation of standard anesthetic care. Adsorption was initiated parallel to skin incision.

Following sternotomy, the internal thoracic artery and simultaneously the saphenous vein harvesting was performed. Prior to the bypass anastomosis, another 15,000 units of heparin were administered (200 IE Heparin/KG body weight with activated coagulation time > 300 s). Myocardial revascularization was performed using an Octopus^®^ tissue stabilizer (Medtronic, Minneapolis, MN, USA) starting with the LITA to the left anterior descending artery in all patients. In one patient, the right internal thoracic artery was grafted to the circumflex artery. For all other bypass grafts, the saphenous vein was used. After the completion of all bypass anastomoses, protamine was given and hemoadsorption was discontinued. Transit-time flow measurement was performed thereafter. The procedure was finished using standard techniques. Patients were then transferred to the intensive care unit (ICU).

### Outcome measures

Primary outcome was the incidence of bleeding complications according to the Bleeding Academic Research Consortium (BARC) [13]. Secondary outcomes included 24-h chest tube drainage (CTD), blood product transfusions, re-operation for bleeding and in-hospital mortality.

## Results

A total of 10 consecutive patients were included. Mean age was 66.7 ± 9.3 years and most of the patients were male (8/10). Demographics are listed in Table 1. A total of 8 patients were treated with ticagrelor preoperatively, one patient was on rivaroxaban and one patient was on both ticagrelor and rivaroxaban. This patient had an acute coronary syndrome with dissection of the LAD and chronic atrial fibrillation. In addition all patients were on aspirin. The OPCAB occurred before the recommended washout period in all patients (AT drug discontinued on the day before surgery in 9 and on the day of surgery in 1 patient). Five patients had coronary stent implantation within the previous 3 months. The OPCAB procedure was completed successfully in all 10 patients and procedural characteristics are summarized in Table 2. Only one BARC-4 bleeding event occurred that required 6 units of red blood cells within the first 48 h postoperatively. The single BARC-4 bleeding event was related to a multimorbid 74-year old female patient on ticagrelor and aspirin, presenting with peripheral vascular disease, previous percutaneous coronary intervention (PCI) of the LAD, chronic kidney injury requiring dialysis, and a stroke, and importantly, a low preoperative hemoglobin (maximum preoperative value of 10.6 g/dL). This patient had an eventful postoperative course without any overt evidence of bleeding and cumulative chest tube drainage of only 200mL, however, she received 6 units of red blood cells to maintain adequate hemoglobin levels within the first 48 h and as such fulfilled the criteria for a BARC-4 bleeding event.

**Table 1:** Demographics

Variable	OPCAB + HA (*n* = 10)
Age, years	67.4 ± 9.2
Gender, male	8 (80)
Prior PCI	5 (50)
Emergency indication (< 24 h)	1 (10)
Urgent indication	9 (90)
Acetylsalicylic acid	10 (100)
Ticagrelor	8 (80)
Rivaroxaban	1 (10)
Ticagrelor + rivaroxaban	1 (10)
NYHA functional class III/IV	10 (100)
Hypertension	9 (90)
Diabetes (NIDDM/IDDM)	4/1 (34.2)
Hyperlipidaemia	10 (100)
History of smoking	6 (60)
Renal dysfunction (creatinine ≥ 1.3 mg/dL / failure (dialysis)	2 (80)
Ejection fraction normal	8 (20)
Ejection fraction moderate impaired (40%; 45%)	2 (20)
BMI	28,8 ± 4,3
BMI > 30	3 (30)
EuroSCORE II, %	2.9 ± 1.5

Data are presented as number (%) or mean±SD; h, hours; OPCAB, off-pump coronary artery bypass grafting; HA, hemoadsorption; NYHA, New York Heart Association; EuroSCORE, European System for Cardiac Operative Risk Evaluation; NIDDM, non-insulin dependent diabetes mellitus; IDDM, insulin dependent diabetes mellitus; PCI, percutaneous coronary intervention; BMI, body mass index

**Table 2:** Procedural outcomes

Procedural outcomes	OPCAB + HA (*n* = 10)
Skin-to-skin time, min	184 ± 35.0
HA duration, min	143 ± 26.6
Number of grafts	2.3 ± 0.9
BARC-4 bleeding	1 (10.0)
>5 RBCs / 48 h	1 (10.0)
Washout period, days	1.4 ± 0.8
RBC (u)	2.7 ± 1.9*
Surgical re-exploration within 7 days	0 (0)
12-hour CTD, mL	540 ± 345*
24-hour CTD, mL	680 ± 307*
Time on ICU, days	6.1 ± 4.8
Hospitalization postoperatively, days	14.6 ± 4.6
Mortality	0 (0)

Data are presented as number (%) or mean±SD; min., minutes; h, hours, d, days; HA, hemoadsorption; BARC, bleeding academic research consortium; RBC, red blood cell; CTD, chest tube drainage; *patient with BARC-4 bleeding excluded

The mean 24-hour CTD volume of the overall cohort was 680 ± 246mL. No surgical re-explorations for bleeding occurred within the index hospitalization. During follow-up one patient underwent a late subxiphoidal evacuation of a pericardial effusion at 2.3 weeks, and all 10 patients were alive at a mean of 19.5 ± 17.0 months without any need for reintervention. In the initial four patients a conventional dialysis pump was used and thereafter the PUR-01 apheresis machine was used.

## Discussion

Herein, a novel innovative approach is described in which a hemadsorption cartridge integrated into an apheresis pump was used for the removal of ticagrelor and rivaroxaban during OPCAB. The main observations from the current study are that AT drug removal via hemoadsorption during OPCAB surgery using a stand-alone apheresis pump was feasible and safe without any device-related events. Moreover, bleeding complications according to the BARC-4 definition were infrequent and overall CTD volumes were modest, suggesting that this maybe an effective intervention to limit perioperative bleeding in these high-risk patients. The current study is the first report of active AT drug removal in patients undergoing off-pump cardiac surgery.

Previously, Tripathi et al. [14] showed in an *in-vitro* study that the hemoadsorption adsorber incorporated into a clinical-scale benchtop recirculation circuit was capable of efficiently removing apixaban, rivaroxaban, and ticagrelor. Early clinical experience with the integration of the adsorber on CPB has also been promising as reported by Hassan et al. [5, 6]. in patients on ticagrelor or rivaroxaban undergoing on-pump CABG or aortic dissection before the recommended washout period was complete. Hassan et al. could show clinical benefits for AT removal including lower incidence of postoperative surgical re-explorations, significantly less blood product transfusions and lower chest tube drainage. The above benefits together with a shortened hospital stay translated into significant cost savings. However, so far, the clinical use in cardiac surgery has been limited to on-pump cases only with the necessary integration into the CPB circuit. Therefore, the current report extends to an entirely new patient population.

An initial case report from our group published in 2020 described a patient presenting with a dissection of the left anterior descending (LAD) artery following a complicated PCI procedure [12]. As the patient was loaded with ticagrelor and was also treated with rivaroxaban due to atrial fibrillation, the patient refused further treatment. However, on the following day, the patient presented with an increase in cardiac troponin-T accompanied by acute chest pain. Finally, the patient agreed to the recommended OPCAB procedure. Before surgery in this *`index´* case, we initiated CytoSorb® adsorption with the adsorber used in a conventional dialysis extracorporeal circuit in hemoperfusion mode without fluid removal. From then on, to save further time and to avoid putting patients at risk by delaying surgery, we started to use intraoperative hemoadsorption during OPCAB procedures directly after skin incision. Our initial experience with this approach including a detailed technical description has been already published and the present analysis summarizes our total experience with intraoperative hemoadsorption during OPCAB surgery using the PUR-01 apheresis machine.

**Fig. 2:**
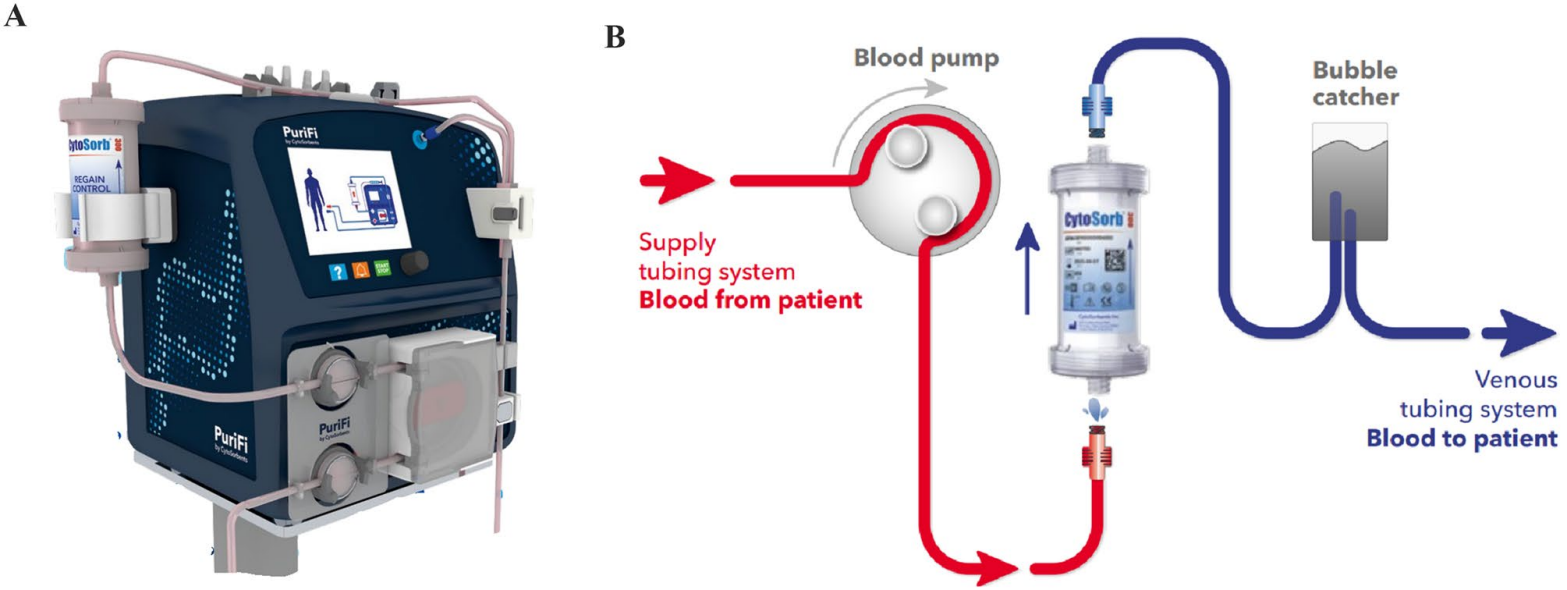
**A**: Hemoadsorption device (CytoSorb® 300, CytoSorbents Inc., Princeton, NJ, USA) incorporated in the apheresis machine (PuriFi by CytoSorbents CytoSorbents Europe GmbH, Germany); **B**: Schematic drawing of the extracorporeal circuit of the PuriFi

In the present analysis two different stand-alone platforms for hemoperfusion have been evaluated. The conventional dialysis system set in hemoperfusion mode seemed to be quite complex to handle and needed continuous monitoring especially with higher flow rates over 120mL/min. Compared to this, the PUR-01 is easy to handle and tolerates higher flow rates without any pressure problems. However, the disadvantage of the PUR-01 is the missing heat exchanger compared to a dialysis setup. Especially during OPCAB surgery, temperature regulation and warming is vital. It is important to note that not only AT’s bear the risk of increased bleeding complications, but also a temperature decrease during surgery or surgery-induced inflammation might influence the coagulation system. Meanwhile a new stand-alone hemoperfusion system has been announced (PuriFi, Medica S.P.A, Medolla, Italy; Fig. 2). This new device offers not only a user-friendly setup covering a wide flow range from 5 to 450mL/min., but importantly also offers the possibility of temperature regulation.

In the current literature, bleeding complications in patients on dual anti-platelet therapy (DAPT) involving ticagrelor and acetylsalicylic acid undergoing isolated CABG surgery within 24-hours after drug discontinuation, report a BARC-4 bleeding rate up to 38% and 24-hour CTD of 813 ± 554mL, resulting in the need for surgical re-exploration within 48 h of 6.1% (4). For our patient series, we have no historical OPCAB control group for comparison. Before the ability to hemoadsorb anti-coagulant substances, patients on these drugs had to wait for the washout period and were operated under CPB conditions, as the reasons for the decision to operate off- or on-pump were in general considered secondary at the time. Nevertheless, at least we could compare our observed CTD volumes of 680mL to a series published by Hassan and coworkers [5]. Hassan et al. compared their results using intraoperative hemoadsorption in 32 ticagrelor patients to a control group of 11 ticagrelor patients without intraoperative hemoadsorption. The controls exhibited an overall CTD volume of 890mL, which is a 30% increase compared to the findings of the present analysis.

The major observation of the present evaluation is that the PUR-01 pump allows the adsorber to be used in patients who do not require CPB, an approach that could potentially also allow active AT removal in patients requiring urgent non-cardiac surgery. Therefore, the decoupling of the adsorber from the CPB circuit could open new future indications in many specialties, and a recent case report published by Dalmastri et al. [15] described the successful removal of apixaban preoperatively in an emergency patient scheduled for bilateral nephrostomy. The authors showed that by adding an adsorber into a conventional renal replacement circuit, apixaban levels were reduced by 48.2% after 2.5 h. The following surgery after apixaban removal was without any complications.

It also should be acknowledged that the CytoSorb® 300 mL device was initially designed to remove cytokines and to attenuate the so-called cytokine storm [16]. Meanwhile, drug elimination through active removal has been extensively evaluated [9].

Of note, the length of ICU stay in the current analysis appears marginally prolonged at 6.1 ± 4.8 days. This is in part explained by the fact that we combined the intermediate care unit stay with the intensive care stay. In addition, clinical reasons other than postoperative bleeding contributed to longer stays in three patients (two patients with active COVID-19 requiring isolation and an additional patient with prolonged delirium). Finally, another two patients were kept for additional days on the intermediate care unit due to signs of mild pulmonary congestion. Notably, in the current series which included 10 patients, no serious adverse device-related events were observed. Moreover, the risk of placing a dialysis catheter would be disproportionate to the intended objective of mitigating bleeding complications by hemoadsorption in patients on antithrombotics undergoing major surgery. Meanwhile, intraoperative hemadsorption has been mentioned by the most recent European Society of Anaesthesiology and Intensive Care (ESAIC) Guidelines for the management of severe perioperative bleeding with a class 2 C indication in patients on ticagrelor or rivaroxaban undergoing emergency cardiac/aortic surgery on cardiopulmonary bypass to reduce bleeding complications [17].

## Conclusion

In conclusion, these first in-human results confirm that AT removal by hemoadsorption appears to be a feasible, safe and effective approach in reducing perioperative bleeding in patients undergoing urgent OPCAB surgery. However, since the current analysis represents a case series, results should be evaluated in future larger trials.

### Limitations

The present analysis is limited by low patient numbers without a control group and the data is derived from a single institution, however, it represents the first large cases series supporting the feasibility of this novel therapeutic intervention. Future prospective trials are required to determine and establish the potential clinical benefits of this application. In on-pump CABG surgery, the pivotal, double blind, randomized Safe and Timely Antithrombotic Removal – Ticagrelor (STAR-T) trial in the US and Canada (ClinicalTrials.gov Identifier: NCT04976530) is evaluating whether intraoperative ticagrelor removal may reduce serious perioperative bleeding complications in patients undergoing surgery before completing the recommended washout period. Also, detailed coagulation parameters or drug concentrations have not been evaluated. In seven patients the PUR-01 device was used without the possibility of temperature control. Following evaluations in OPCAB surgery hemoperfusion platforms should be used when there is the possibility of temperature control. Finally, future evaluations should focus on the optimal flow for drug removal and potential postoperative continuation.

## Data Availability

The authors confirm that the data supporting the findings of this study are available within the article and will be shared upon request by the corresponding author.

## Abbreviations

AT: Antithrombotic
BARC: Bleeding Academic Research Consortium (BARC)-4
CABG: Coronary Artery Bypass Grafting
CPB: Cardiopulmonary Bypass
CTD: Chest Tube Drainage
CVVHD: Continuous Veno-Venous Hemodiafiltration
DAPT: Dual Anti-Platelet Therapy
DOAC: Direct Oral Anticoagulants
ESAIC: European Society of Anaesthesiology and Intensive Care
ICU: Intensive Care Unit
LAD: Left Anterior Descending (artery)
LITA: Left Internal Thoracic Artery
MACCE: Major Adverse Cardiac and Cerebral Events
OPCAB: Off-Pump Coronary Artery Bypass
PCI: Percutaneous Coronary Intervention
